# Association of licensure and relationship requirement waivers with out-of-state tele-mental health care, 2019–2021

**DOI:** 10.1093/haschl/qxae026

**Published:** 2024-02-28

**Authors:** Antonios M Koumpias, Owen Fleming, Lewei Allison Lin

**Affiliations:** Department of Social Sciences, University of Michigan-Dearborn, Dearborn, MI 48128, United States; Department of Economics, Wayne State University, Detroit, MI 48202, United States; Addiction Center, Department of Psychiatry, University of Michigan Medical School, Ann Arbor, MI 48109, United States; Veterans Affairs Center for Clinical Management Research, Veterans Affairs Ann Arbor Healthcare System, Ann Arbor, MI 48105, United States

**Keywords:** out-of-state tele-mental healthcare, in-state licensure requirements, pre-existing patient-physician relationship requirement, interstate medical licensure compact, COVID-19 public health emergency, COVID-19 research database

## Abstract

During the COVID-19 public health emergency, states waived in-state licensure and pre-existing patient–physician relationship requirements to increase access to care. We exploit this state telehealth policy variation to estimate the association of in-state licensure requirement waivers and pre-existing patient–physician relationship requirement waivers with out-of-state tele-mental health care utilization of patients diagnosed with COVID-19. Using claims from January 2019 until December 2021 of 2 037 977 commercially insured individuals in 3 metropolitan statistical areas (MSAs) straddling Midwestern state borders, we found increased out-of-state telehealth utilization as a share of out-of-state mental health care by 0.1411 and 0.0575 visits per month or 1679.76% and 467.48% after licensure and relationship waivers, respectively. Within-MSA analyses illustrate an urban–rural digital divide in out-of-state utilization as a share of total or telehealth mental health care. Our findings indicate waivers primarily enhance access to care of established patients by enabling the transition of in-person out-of-state health care online. Interstate medical licensure compact participation may provide broader access to out-of-state tele-mental health care than emergency waivers.

## Introduction

The COVID-19 public health emergency (PHE) has exerted a significant toll on the mental health of individuals in the United States, with nearly 1 in 3 young adults reporting any mental illness in 2021.^1^ The mental well-being of individuals diagnosed with COVID-19 may be particularly strained due to loneliness precipitated by physical distancing and stay-at-home or pre-existing challenges to accessing mental health care.^2-5^ Telehealth is particularly promising for mental health treatment; prior to the PHE, mental health comprised the majority of telehealth visits due to the increased feasibility of telehealth delivery of mental health care, which focuses less on physical assessments and interventions.^6-8^ While tele-mental health care has experienced tremendous growth following the broad emergency waivers granted during the PHE, the effect of individual telehealth flexibilities on out-of-state tele-mental health care utilization of commercially insured individuals remains unknown.

Out-of-state health care is of paramount importance for patients residing in health professional shortage areas to access specialty services, including mental health care, and mitigate shortages in PHE hotspots.^9,10^ In-state licensure requirement waivers (licensure waivers) represent state-issued policy changes during the PHE that allow providers to deliver tele-mental health care beyond their licensed states. In the presence of large unmet needs, this mechanism of licensure portability may alleviate health care shortages by providing access to out-of-state health care for patients near state borders.

Alongside licensure waivers, some states also waive the pre-existing patient–physician relationship requirement (relationship waivers), permitting providers to establish new patient–physician relationships via telehealth. Increased salience of this flexibility coupled with reduced oversight through the elimination of pre-existing relationship audits may enhance clinician and patient interest in establishing new relationships via telehealth. Therefore, we expect that relationship waivers will significantly enhance out-of-state utilization by new patients. However, for patients residing in states participating in the Interstate Medical Licensure Compact (IMLC), where availability of out-of-state providers is already prevalent, the influence of relationship waivers on out-of-state tele-mental health care may not be as pronounced.

Previous research on out-of-state telehealth utilization during the PHE has either focused on Medicare beneficiaries or evaluated the broad effects of PHE telehealth flexibilities ranging from audiovisual modality and originating site requirements, or mandatory reimbursement rules such as payment parity.^11-14^ This work builds on these studies by decomposing the aggregate effect of telehealth policy changes to isolate the impact of relationship waivers from changes associated with licensure waivers. We focus on out-of-state tele-mental health care of commercially insured minors and adults up to age 64 years who live near a state border and were diagnosed with COVID-19. We used a COVID-19 patient database in this study, which provides granular geolocation information that allowed us to perform within–metropolitan statistical area (-MSA) comparisons. We considered 3 Midwestern MSAs straddling state borders that exhibit policy variation in relationship waivers: the Chicago-Naperville-Joliet MSA (Chicago MSA) in Illinois-Indiana-Wisconsin, the Davenport-Moline-Rock Island MSA (Davenport MSA) in Iowa-Illinois, and the St. Louis MSA in Missouri-Illinois. We compared the pre- and post-reform monthly rates of out-of-state tele-mental health care in Iowa, Indiana, Missouri, and Wisconsin, where licensure and requirement waivers are enacted to Illinois where only licensure waivers are extended, using a difference-in-differences (DID) model and conducted event study falsification tests. Within-MSA comparisons provide an ideal setting to estimate the maximum reach of out-of-state telehealth utilization by patients residing near state borders while accounting for health shocks commonly affecting cross-border communities.

In addition, we investigated whether the impact of the relationship waiver varies across new and established patients, participation in the IMLC, or level of urbanization. Our findings provide state policymakers with more nuanced estimates of the role of a distinct telehealth policy change in increasing access to out-of-state tele-mental health care relative to licensure waivers.

## Data and methods

We used Change Healthcare claims data provided by the COVID-19 Research Database. Although exclusively focused on patients with COVID-19, this dataset, with its granular geographic identifiers, is well suited for our border discontinuity design.^15^ Each claim in the dataset identifies a unique visit. We collapsed services onto the claim level, denoting a visit, to track the number of mental health visits for each patient in a given month, our unit of analysis (Appendix Table A1). This study was deemed nonregulated and exempt from review by the University of Michigan Institutional Research Board under contract HUM00238506.

Our study population included all patients under 65 years from 3-digit zip codes within the Chicago, Davenport, or St. Louis MSAs, who are covered by commercial insurance, recorded at least 1 in-person outpatient mental health visit to a provider within the 3 MSAs in 2019, and had a laboratory-confirmed COVID-19 diagnosis between January 1, 2019, and December 31, 2021 (Appendix Table A2). Mental health diagnoses and evaluation and management (EM) visits are identified by International Classification of Diseases, Ninth and Tenth Revision (ICD-9/10), Healthcare Common Procedure Coding System (HCPCS) and Current Procedural Terminology (CPT) codes, following the literature (Appendix Table A1).^16-18^ We removed visits where patient status (new or established) cannot be determined (inclusion/exclusion criteria in Appendix Figure A1). Our outcomes of interest were out-of-state tele-mental health care measured as a share of (1) all mental health visits, (2) all telehealth mental health visits, or (3) all out-of-state mental health visits per patient-month.

First, we examined changes in total mental health visits by modality and physician location. Second, we provided summary statistics of all study variables and calculated unadjusted rates of out-of-state telehealth utilization. Then, we used a DID model to measure how telehealth reform changes the rates of out-of-state tele-mental health care. We estimated the association between licensure waivers and out-of-state telehealth utilization rates by computing an adjusted difference of the outcomes for all states between the pre- and post-waiver period. This difference is captured by the coefficient on the time-specific indicator that denotes the first full month that licensure waivers are in place, April 2020. Then, exploiting policy variation in both time and space, we identified the impact of the relationship waivers by comparing the change in out-of-state telehealth utilization of patients in Indiana, Iowa, Missouri, and Wisconsin between the pre- and post-waiver periods with the change in outcomes of patients in Illinois within the 3 MSAs of interest (Appendix Figure A2).^19^ The parameter of interest is the estimated coefficient of the interaction between the time-specific waiver indicator and state-specific waiver indicators representing the introduction of relationship waivers in Indiana, Iowa, Missouri, and Wisconsin. We adjusted our baseline results with individual-level variables such as age, gender, and commercial insurance type and controlled for time-invariant unobservable local area characteristics through 3-digit zip code fixed effects. In sensitivity analysis, we used demographic and socioeconomic 2020 US Census information at the 3-digit zip code level, such as population, percentage Black, unemployment rate, and 14 other characteristics, to account for community-level differences that may impact tele-mental health care and specified state fixed effects (Appendix Table A3). To assess the validity of our design, we conducted event study falsification tests using either March 2020 or February 2020 as the baseline pre-waiver month. Finally, we stratified by MSA to uncover potential mechanisms driving out-of-state health care utilization rates, such as state IMLC participation or county urbanization level (Appendix Table A3). The analysis was performed using statistical software (Stata 17/SE; StataCorp, College Station, TX), and at the 10%, 5%, and 1% significance level. This study follows the Strengthening the Reporting of Observational Studies in Epidemiology (STROBE) reporting guidelines for cross-sectional studies.

## Results

### Study population

We identified a total of 12 322 468 mental health visits (24.90% IN, IA, MO, and WI; 75.10% IL) from January 1, 2019, through December 31, 2021, by 2 029 470 patients (26.04% IN, IA, MO, and WI; 73.96% IL) who were diagnosed with COVID-19 and had at least 1 mental health visit in 2019 (Table 1). On average (SD), patients in Indiana, Iowa, Missouri, and Wisconsin recorded 1.3340 (0.7751) monthly mental health visits, of which 6.26% were delivered via telehealth and 1.13% via telehealth by an out-of-state provider, whereas patients in Illinois accessed mental health services 1.352 (0.8312) times per month, 7.96% of which were via telehealth but only 0.31% via telehealth at an out-of-state provider. Further, robust in-person, out-of-state mental health visits were reported, representing 10% and 5.6% of mental health care in treated and control states, respectively. In this predominantly female sample, the percentage of male patients and age profile in treated and control states was similar. Illinois patients were more likely to be covered by BlueCross/BlueShield or managed care plans than those in treated states.

**Table 1.** Summary statistics for treated and control states, 2019–2021.

	Treated states (IN, IA, MO, WI)				Control state (IL)			
	Mean	SD	Min	Max	Mean	SD	Min	Max
Monthly visits								
MH visits	1.3340	0.7751	1	52	1.3520	0.8312	1	64
In-person in-state MH visits	1.1171	0.8071	0	44	1.1684	0.8567	0	54
Telehealth in-state MH visits	0.0683	0.3110	0	25	0.1036	0.4121	0	44
In-person out-of-state MH visits	0.1335	0.4982	0	52	0.0758	0.3531	0	64
Telehealth out-of-state MH visits	0.0151	0.1745	0	20	0.0041	0.0783	0	12
Demographics								
Male	0.4000	0.4899	0	1	0.4046	0.4908	0	1
Age: minor	0.2137	0.4099	0	1	0.2684	0.4431	0	1
Age: 18–34 y	0.1934	0.3949	0	1	0.1957	0.3967	0	1
Age: 35–49 y	0.2391	0.4265	0	1	0.2158	0.4114	0	1
Age: 50–64 y	0.3539	0.4782	0	1	0.3201	0.4665	0	1
Insurance status								
Commercial insurance	0.8705	0.3357	0	1	0.5585	0.4966	0	1
BCBS	0.0938	0.2916	0	1	0.3641	0.4812	0	1
PPO	0.0209	0.1432	0	1	0.0223	0.1478	0	1
EPO	0.0003	0.0184	0	1	0.0004	0.0199	0	1
HMO	0.0144	0.1191	0	1	0.0547	0.2274	0	1

	Treated states (IN, IA, MO, WI)				Control state (IL)			
	Pre-waiver period		Post-waiver period		Pre-waiver period		Post-waiver period	
Outcome counts and proportions								
Total MH visits	1 872 461		1 196 057		5 149 875		4 104 075	
% MH visits that are OOS telehealth	0.1873%		2.6121%		0.0398%		0.6406%	
Telehealth MH visits	41 812		150 156		27 810		709 514	
% Telehealth MH visits that are OOS	8.3875%		20.8064%		7.3714%		3.7054%	
OOS MH visits	162 296		179 438		311 134		236 196	
% OOS MH visits that are telehealth	2.1609%		17.4110%		0.6589%		11.1306%	
% In-person MH visits that are OOS	8.6739%		14.1692%		6.0344%		6.1836%	
Totals								
Observations	2 300 272				6 844 680			
Patients	530 621				1 507 356			
MH visits	3 068 518				9 253 950			

Abbreviations: BCBS, Blue Cross/Blue Shield; EPO, Exclusive Provider Organization; HMO, Health Maintenance Organization; IA, Iowa; IL, Illinois; IN, Indiana; Max, maximum; MH, mental health; Min, minimum; MO, Missouri; OOS, out-of-state; PPO, Preferred Provider Organization; WI, Wisconsin.

Source: Authors’ analysis of the Change Healthcare dataset provisioned by the COVID-19 Research Database, 2019–2021. This table reports summary statistics of tele-mental health care utilization in the treated (IN, IA, MO, WI) states introducing both licensure and relationship waivers and the control (IL) state granting the licensure waiver only and across pre- and post-treatment periods over the sample period 2019–2021. Numbers for monthly visits are computed by first aggregating visit-level information on modality and 3-digit zip code location to a monthly count for each individual, then summarizing the monthly counts. We also report summary demographic and insurance status information. We report baseline outcome measures of total visits by modality and location, as well as percentage of visits by modality and location, for the treated and untreated states in the pre- and post-waiver period. The pre-waiver period for the treated states IA and IN is defined as all months before April 2020, for MO as all months before July 2020, and for WI as all months before April 2020, as well as June 2020 through September 2020. The pre-waiver period for the control state IL is defined as all months before April 2020. These numbers are computed by aggregating visits by modality and location to the state level for the pre- and post-periods, then taking percentages. The sample includes 2 029 470 distinct patients selected according to the inclusion-exclusion criteria outlined in Appendix Figure A1. Note that the sum of distinct patients in the 2 sets of states (2 037 977) exceeds the total number of distinct patients as we allow for the possibility that patients move between full and partial-waiver states.

**Figure 1.**
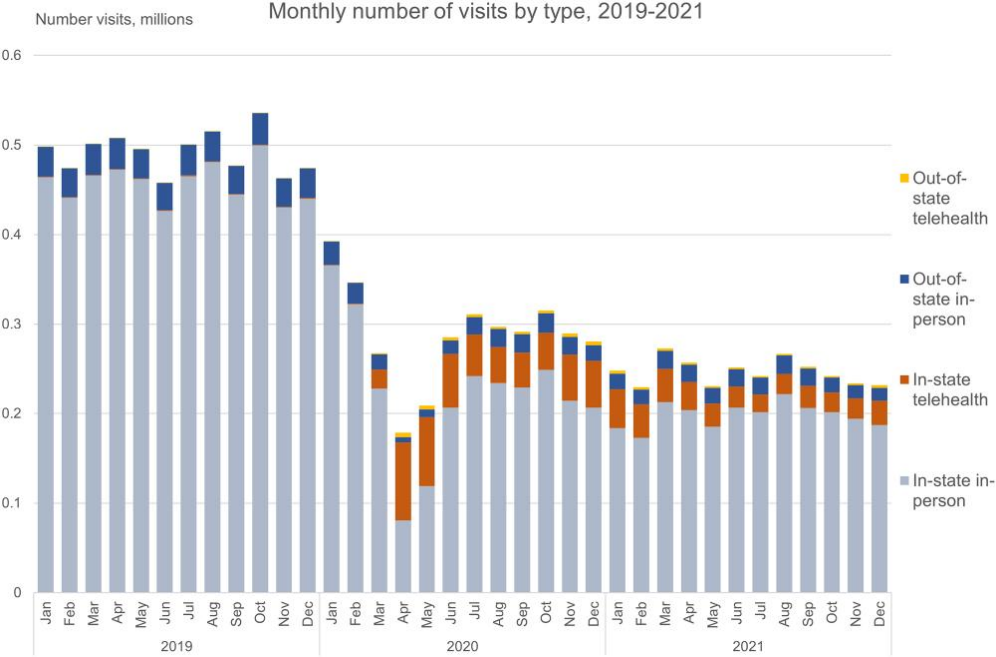
Monthly number of visits by type, 2019–2021. This figure depicts the total number of visits type by month during the 2019–2021 study period. For each visit, we identified whether it was in-state in-person, in-state telehealth, out-of-state in-person, or out-of-state telehealth and then aggregated visit-level information to the year-month level. Source: Authors’ analysis of the Change Healthcare dataset provisioned by the COVID-19 Research Database, 2019–2021.

Figure 1 illustrates trends in mental health utilization by the total number of in-state in-person, in-state telehealth, out-of-state in-person, and out-of-state telehealth mental health visits from January 1, 2019, through December 2021 in monthly frequency. Utilization peaked at historically high levels in April 2020 when more than 51.42% of mental health visits were delivered via telehealth, thereby eclipsing in-person mental health care for the first time.^20^ Results indicate extensive deferral of mental health care during the PHE relative to 2019, driven by reductions of in-state, in-person care commencing in January 2020. In- or out-of-state telehealth utilization did not revert to pre-PHE levels after the initial wave of COVID-19 but instead remained elevated for at least 20 months following the initial declaration of the PHE. A list of the 10 most common clinical procedures based on CPT codes is displayed in Appendix Table A4.

### Licensure waiver association with out-of-state tele-mental health care

Table 2 presents this study's baseline findings. After licensure waivers were adopted by all states, out-of-state telehealth utilization as a share of total mental health visits rose by 1680% (estimate: 0.0084; 95% CI: 0.0029–0.01388), driven by strong increases in new patient visits. There was a 113.80% (estimate: −0.1229; 95% CI: −0.0682 to −0.1775) decrease in out-of-state telehealth visits as a share of telehealth mental health visits, which may be attributed to the steep rise in overall telehealth utilization during the PHE (Figure 1). Licensure waivers were associated with increased out-of-state tele-mental health care as a share of out-of-state mental health visits by 1679.76% (estimate: 0.1411; 95% CI: 0.0147 to 0.2675) relative to pre-PHE utilization.

**Table 2.** Change in out-of-state tele-mental health care visits after licensure and relationship waivers.

	Share of total visits			Share of telehealth visits			Share of out-of-state visits		
	(1) All patients, visits (95% CI)	(2) New patients, visits (95% CI)	(3) Established patients, visits (95% CI)	(4) All patients, visits (95% CI)	(5) New patients, visits (95% CI)	(6) Established patients, visits (95% CI)	(7) All patients, visits (95% CI)	(8) New patient, visits (95% CI)	(9) Established patient, visits (95% CI)
Post	0.0084**	0.0457**	0.0043*	−0.1229**	−0.3677***	−0.0514*	0.1411*	0.3226*	0.0902**
	(0.0029, 0.0139)	(0.0147, 0.0767)	(0.0012, 0.0074)	(−0.1772, −0.0686)	(−0.4183, −0.3171)	(−0.0916, −0.0112)	(0.0149, 0.2673)	(0.0769, 0.5683)	(0.0269, 0.1535)
Treat	0.0011	0.0031	0.0009	0.1456**	0.1905	0.1205**	−0.0085	0.0027	−0.0101
	(−0.0071, 0.0093)	(−0.0011, 0.0073)	(−0.0080, 0.0098)	(0.0571, 0.2341)	(−0.0395, 0.4205)	(0.0619, 0.1791)	(−0.0243, 0.0073)	(−0.0029, 0.0083)	(−0.0295, 0.0093)
Post × Treat	0.0118	0.0019	0.0129	−0.0059	0.0987	0.0144	0.0575**	−0.004	0.0677**
	(−0.0061, 0.0297)	(−0.0083, 0.0121)	(−0.0083, 0.0121)	(−0.0998, 0.0880)	(−0.1595, 0.3570)	(−0.0474, 0.0762)	(0.0210, 0.0940)	(−0.0153, 0.0073)	(0.0243, 0.1111)
ȳ	0.0005	0.0017	0.0004	0.108	0.6484	0.0727	0.0084	0.019	0.0063
ȳ, tr	0.001	0.0028	0.0007	0.207	0.6977	0.1477	0.0123	0.027	0.0094
*n*	9 144 952	1 231 017	8 239 083	723 290	32 272	696 474	661 191	114 988	567 391

Abbreviation: *n*, no. of observations; ȳ, Outcome mean; ȳ, tr, Outcome mean in treated states.

Source: Authors’ analysis of the Change Healthcare dataset provisioned by the COVID-19 Research Database, 2019–2021. The table presents the difference-in-differences regression estimates (95% CI in parentheses) of the association of licensure and relationship waivers with out-of-state tele-mental health care. The sample includes mental health and evaluation and management visits of patients in Illinois, Indiana, Iowa, Missouri, and Wisconsin over 2019–2021. For columns (1)–(3), the outcome is the share of all mental visits that are out-of-state telehealth; for columns (4)–(6), the share of telehealth visits that are out-of-state; and for columns (7)–(9), the share of out-of-state visits that are telehealth. Columns (1), (4), and (7) consider all patient visits; (2), (5), and (8) consider new patient visits; and (3), (6), and (9) consider established patient visits. In all relationship-specific columns, denominators of the outcome variables are themselves relationship-specific—for example, the outcome for column (2) is the total number of new patient out-of-state telehealth mental health visits divided by the total number of new patient mental health visits. Post is an indicator variable taking values equal to 1 from April 2020 onwards; 0, otherwise, and its coefficient estimate captures the association of the licensure waivers with out-of-state tele-mental care. Treat is an indicator for Indiana, Iowa, Missouri, and Wisconsin. The coefficient estimate of the interaction term Post × Treat represents the association of the relationship waivers with out-of-state tele-mental care. Regressions include zip code and month fixed effects and gender, age, and insurance status whose estimates are omitted for brevity. Standard errors are clustered by state. *P < 0.10; **P < 0.05; ***P < 0.01.

### Relationship waiver effect on out-of-state tele-mental health care

The estimated DID coefficient revealed no significant association between the relationship waiver on out-of-state telehealth utilization as a share of total or telehealth-only mental health visits. The relationship waiver resulted in an expansion in the share of out-of-state visits conducted via telehealth by 467.48% (estimate: 0.0677; 95% CI: 0.0248–0.1106). We estimated a 720.21% increase in utilization by established patients, but no significant association between the relationship waiver and new patient visits, the policy's intended target group (Appendix Figure A3). Results were robust to alternative specifications that used state fixed effects with 3-digit zip code covariates or considered March 2020 as the licensure implementation month (Appendix Tables A5 and A6). The event study plots indicate the absence of any large differences in pre-waiver outcome trends between the treated and control states, lending support to the DID model (Appendix Figures A4–A6). Analyses by age groups (minors, 18–34, 35–49, 50–64 years) did not reveal patterns that are qualitatively different to our baseline results and suggest that the 18–34-year group has the highest utilization increases (Appendix Figure A7).

### Licensure waiver association with out-of-state tele-mental health care by MSA

In the Chicago MSA, licensure waivers were associated with a rise in out-of-state tele-mental health care as a share of total mental health utilization of new patients by 2117.86% (estimate: 0.0593; 95% CI: 0.0281–0.0905) and a reduction in out-of-state tele-mental health care as a share of total tele-mental health care by 57.38% (estimate: −0.3460; 95% CI: −0.2415 to −0.4505) for new patients. In the Davenport MSA, licensure waivers were correlated with reductions in out-of-state telehealth visits as a share of total tele-mental health care by 55.87% (estimate: −0.4601; 95% CI: −0.4395 to −0.4807) for new patients and increases in out-of-state telehealth visits as a share of total out-of-state mental health utilization by 326.63% (estimate: 0.0601; 95% CI: 0.0519 to 0.0683) for established patients. In the St. Louis MSA, we found reductions in out-of-state telehealth visits as a share of total tele-mental health care of new patients by 115.89% (estimate: −0.8658; 95% CI: −1.0387 to −0.6929) and increases in out-of-state telehealth visits as a share of total out-of-state mental health utilization for all patients by 237.13% (estimate: 0.0728; 95% CI: 0.0595 to 0.0861), driven by increased utilization of established patients by 402.36% (estimate: 0.0853; 95% CI: 0.0779–0.0927).

### Relationship waiver effect on out-of-state tele-mental health care by MSA

In the Chicago MSA, relationship waivers were estimated to increase out-of-state telehealth utilization as a share of total mental health utilization by 2100% (estimate: 0.0315; 95% CI: 0.0156–0.0474), or by 2584.62% (estimate: 0.0336; 95% CI: 0.0164–0.0508) for established patients, and by 425% (estimate: 0.0119; 95% CI: 0.0068–0.0170) for new patients (Table 3). Relationship waivers did not contribute to an increase in out-of-state tele-mental visits as a share of tele-mental health care for all patients, despite an increase of 57.13% (estimate: 0.3445; 95% CI: 0.1871–0.5019) for new patients in Indiana or Wisconsin relative to new patients in Illinois of the Chicago MSA.

**Table 3.** Change in out-of-state tele-mental health care visits after licensure and relationship waivers by MSA.

	Chicago-Naperville-Joliet MSA			Davenport-Moline-Rock Island MSA			St. Louis MSA		
	(1) All patients, visits (95% CI)	(2) New patients, visits (95% CI)	(3) Established patients, visits (95% CI)	(4) All patients, visits (95% CI)	(5) New patients, visits (95% CI)	(6) Established patients, visits (95% CI)	(7) All patients, visits (95% CI)	(8) New patients, visits (95% CI)	(9) Established patients, visits (95% CI)
Share of total visits									
Post	0.0076	0.0593*	0.0019	0.0228	0.0014	0.0249	0.0152	0.0065	0.0161
	(−0.0007, 0.0159)	(0.0280, 0.0906)	(−0.0042, 0.0080)	(0.0037, 0.0419)	(−0.0046, 0.0074)	(0.0037, 0.0461)	(0.0065, 0.0239)	(0.0032, 0.0098)	(0.0073, 0.0249)
Treat	−0.008	−0.0018	−0.0089	0.0083*	0.0065**	0.0085*	−0.0018	0.0011***	−0.0023*
	(−0.0150, −0.0010)	(−0.0035, −0.0001)	(−0.0164, −0.0014)	(0.0067, 0.0099)	(0.0063, 0.0067)	(0.0067, 0.0103)	(−0.0024, −0.0012)	(0.0011, 0.0011)	(−0.0031, −0.0015)
Post × Treat	0.0315*	0.0119**	0.0336*	−0.0215***	−0.0064***	−0.0224***	−0.0129***	−0.0087**	−0.0131***
	(0.0157, 0.0473).	(0.0068, 0.0170).	(0.0164, 0.0508)	(−0.0215, −0.0215)	(−0.0064, −0.0064)	(−0.0224, −0.0224)	(−0.0133, −0.0125)	(−0.0095, −0.0079)	(0.0002)
ȳ	0.0004	0.0013	0.0003	0.0016	0.0045	0.0013	0.0006	0.0021	0.0004
ȳ, tr	0.0015	0.0028	0.0013	0.0011	0.0043	0.0008	0.0006	0.0025	0.0003
*n*	6 658 614	879 083	6 009 382	662 892	79 174	609 500	1 823 446	272 760	1 620 201
Share of telehealth visits									
Post	−0.0921*	−0.3460**	−0.0403	−0.2369	−0.4601**	−0.138	−0.4484	−0.8658*	−0.2568
	(−0.136, −0.0482)	(−0.450, −0.242)	(−0.0688, −0.0118)	(−0.4866, 0.0128)	(−0.4805, −0.4397)	(−0.4037, 0.1277)	(−0.6162, −0.2806)	(−1.0397, −0.6919)	(−0.3721, −0.1415)
Treat	0.1373	−0.0214	0.1161	−0.1483*	0.2753*	−0.1473*	−0.1271**	−0.0242	−0.1372**
	(0.0384, 0.2362)	(−0.1591, 0.1163)	(0.0300, 0.2022)	(−0.1917, −0.1049)	(0.1971, 0.3535)	(−0.1905, −0.1041)	(−0.1326, −0.1216)	(−0.0475, −0.0009)	(−0.1417, −0.1327)
Post × Treat	0.0238	0.3445*	0.0287	0.1152***	−0.1312**	0.1075**	0.0130**	−0.0196	0.0220**
	(−0.0741, 0.1217)	(0.1872, 0.5018)	(−0.0560, 0.1134)	(0.1120, 0.1184)	(−0.1447, −0.1177)	(0.1022, 0.1128)	(0.0119, 0.0141)	(−0.0843, 0.0451)	(0.0209, 0.0231)
ȳ	0.075	0.5577	0.0496	0.2734	0.8885	0.2179	0.2042	0.7625	0.1269
ȳ, tr	0.2549	0.603	0.2113	0.1437	0.8235	0.0962	0.1867	0.7471	0.1013
*n*	559 418	24 999	538 916	33 327	1510	32 019	130 545	5763	125 539
Share of out-of-state visits									
Post	0.2204	0.4659	0.1426	0.0504	−0.0129	0.0601**	0.0728*	−0.001	0.0853**
	(−0.0195, 0.4603)	(0.0901, 0.8417)	(0.0355, 0.2497)	(0.0337, 0.0671)	(−0.0966, 0.0708)	(0.0519, 0.0683)	(0.0594, 0.0862)	(−0.0641, 0.0621)	(0.0778, 0.0928)
Treat	0.017	0.0061	0.0166	0.0034**	0.0333**	−0.0027*	−0.0222	0.0462**	−0.0449*
	(−0.0093, 0.0433)	(−0.0069, 0.0191)	(−0.0154, 0.0486)	(0.0030, 0.0038)	(0.0314, 0.0352)	(−0.0033, −0.0021)	(−0.0296, −0.0148)	(0.0428, 0.0496)	(−0.0529, −0.0369)
Post × Treat	0.001	−0.0463	0.0072	0.0629**	0.0338**	0.0717**	0.0923**	0.0174**	0.1193**
	(−0.0467, 0.0487)	(−0.0833, −0.0093)	(−0.0502, 0.0646)	(0.0605, 0.0653)	(0.0307, 0.0369)	(0.0690, 0.0744)	(0.0876, 0.0970)	(0.0162, 0.0186)	(0.1138, 0.1248)
ȳ	0.0106	0.0205	0.0081	0.0052	0.0149	0.0043	0.0088	0.0195	0.006
ȳ, tr	0.0081	0.0145	0.0069	0.0231	0.0498	0.0184	0.0307	0.0532	0.0212
*n*	294 865	59 610	246 256	226 614	25 164	207 396	139 712	30 214	113 739

Abbreviations: IMLC, Interstate Medical Licensure Compact; MSA, metropolitan statistical area; *n*, no. of observations; OOS, out-of-state; ȳ, Outcome mean; ȳ, tr, Outcome mean in treated states.

Source: Authors analysis of the Change Healthcare dataset provisioned by the COVID-19 Research Database, 2019–2021. The table presents the difference-in-differences regression estimates (95% CI in parentheses) of the association of licensure and relationship waivers with out-of-state tele-mental health care stratified by MSA to explore how variation in IMLC participation in the treatment and control states influences our findings. Whereas Iowa, Illinois, and Wisconsin have been members of the IMLC since 2015, Indiana and Missouri only joined in 2023, after the end of our study period. This stratification also allows us to investigate how the presence of an in-state, major urban center in an MSA, or lack thereof, influences demand for out-of-state tele-mental health care since there are 2 MSAs with a major urban center in our study sample but residents of only 1 (St. Louis, Missouri) are also able to initiate visits without a pre-existing patient–physician relationship following waivers. For columns (1)–(3), the regression includes only residents of the Chicago-Naperville-Joliet MSA; for columns (4)–(6), it includes only residents of the Davenport-Moline-Rock Island MSA; and for columns (7)–(9), it includes only residents of the St. Louis MSA. Columns (1), (4), and (7) consider all patient visits; (2), (5), and (8) consider new patient visits; and (3), (6), and (9) consider established patient visits. In all relationship-specific columns, denominators of the outcome variables are themselves relationship-specific—for example, the outcome for column (2) is the total number of new patient OOS telehealth mental health visits divided by the total number of new patient mental health visits. Under each column, coefficient estimates and 95% CIs are presented for each of the 3 main outcomes: the first coefficient estimate is for the share of all mental health visits that are OOS telehealth, the second coefficient estimate is for share of telehealth visits that are OOS, and the third coefficient estimate is for the share of OOS visits that are telehealth. Post is an indicator variable taking values equal to 1 from April 2020 onwards; 0, otherwise, and its coefficient estimate captures the association of licensure waivers with out-of-state tele-mental care. Treat is an indicator for Indiana, Iowa, Missouri, and Wisconsin. The coefficient estimate of the interaction term Post × Treat represents the association of relationship waivers with out-of-state tele-mental care. All regressions include zip code and month fixed effects and individual covariates (gender, age, and insurance status), which are omitted from the table. Standard errors are clustered by state. *P < 0.10; **P < 0.05; ***P < 0.01.

In the Davenport MSA, we found reductions in out-of-state telehealth visits as a share of total mental health visits by 1954.55% (estimate: −0.0215; 95% CI: −0.0214 to −0.0216), fewer new patient visits/month by 148.84% (estimate: −0.0064; 95% CI: −0.0063 to −0.0065), and fewer established patient visits/month by 2800% (estimate: −0.0224; 95% CI: −0.0223 to −0.0225) in Iowa relative to Illinois. Also, we found an 80.17% (estimate: 0.1152; 95% CI: 0.1121–0.1183) increase in out-of-state tele-mental health care as a share of total tele-mental health care for Iowa relative to Illinois patients, which is the combination of a 111.75% increase (estimate: 0.1075; 95% CI: 0.1022–0.1128) in established patient visits/month and a 15.93% decrease (estimate: −0.1312; 95% CI: −0.1121 to −0.1183) in new patient visits/month. Following relationship waivers, overall out-of-state telehealth utilization as a share of out-of-state mental health visits increased by 272.29% (estimate: 0.0629; 95% CI: 0.0605–0.0653) or by 67.87% (estimate: 0.0338; 95% CI: 0.0307–0.0369) for new patients and 389.67% (estimate: 0.0717; 95% CI: 0.0690–0.0744) for established patients.

In the St. Louis MSA, we estimated that relationship waivers are associated with a reduction in out-of-state telehealth mental health visits as a share of tele-mental health care by 2150% (estimate: −0.0129; 95% CI: −0.0125 to −0.0133) for all patients, by 348% (estimate: −0.0087; 95% CI: −0.0079 to −0.0095) for new patients, and by 4366.37% (estimate: −0.0131; 95% CI: −0.0127 to −0.0135) for established patients in Missouri relative to Illinois. Patients in Missouri experienced a 6.96% (estimate: 0.0130; 95% CI: 0.0118–0.0142) increase in out-of-state telehealth mental health visits as a share of tele-mental health care to Illinois patients in the St. Louis MSA, driven by an increase in out-of-state telehealth mental health utilization of established patients by 21.72% (estimate: 0.0220; 95% CI: 0.0208–0.0232). After relationship waivers, utilization of out-of-state tele-mental health care as a share of out-of-state health care increased by 300.65% (estimate: 0.0923; 95% CI: 0.0876–0.0970) for all patients, by 562.74% (estimate: 0.01193; 95% CI: 0.1138–0.1248) for established patients, and by 32.71% (estimate: 0.0174; 95% CI: 0.0162–0.0186) for new patients.

## Discussion

By removing regulatory barriers such as in-state licensure and pre-existing patient–physician relationship requirements, proponents of telehealth argue that out-of-state providers may alleviate tele-mental health care shortages by dramatically expanding access points.^21^ We found that licensure waivers during the PHE were associated with very large, approximately 16-fold, increases in access to out-of-state tele-mental health care as a share of total mental health visits and as a share of out-of-state mental health visits but negatively associated with out-of-state tele-mental health care as a share of telehealth mental health visits. These results are consistent with the estimates of a 10-fold increase in tele-mental health care for commercially insured US adults and a 30-fold increase for youths between 2019 and 2022.^13,14^ Following relationship waiver implementation, evidence of increased utilization of tele-mental health care is restricted to out-of-state telehealth as a share of out-of-state mental health visits only. The insignificant association with tele-mental health care as a share of total mental health visits might result from increased utilization in the Chicago MSA, offset by decreased utilization in the Davenport and St. Louis MSAs. Increases in out-of-state telehealth as a share of tele-mental health care in the Davenport and St. Louis MSAs were not sufficiently large to translate to the full sample, potentially due to the insignificant association in the Chicago MSA, in contrast to increases in out-of-state telehealth as a share of out-of-state mental health visits.

Our results are broadly consistent with prior literature documenting the rise in out-of-state telehealth utilization following licensure waivers. Previous work documents out-of-state telehealth utilization rates of 0.1% for all EM visits and 8% of all telehealth visits in 2019 to 0.8% of all EM visits and 5% of all telehealth visits in 2020 or from 4.5% of out-of-state telehealth visits in April 2020 to 5.6% by June 2021.^11,12^ We report rising out-of-state tele-mental visits from 0.04% of all visits in 2019 to 1.1% in 2020 and 0.9% in 2021 and a decrease from 19% of all telehealth visits to 8.4% in 2020 and 7.4% in 2021. Our findings contribute to the ongoing policy discourse on waiver expiration by highlighting the relatively greater importance of out-of-state tele-mental health care for patients residing near state borders.^11,12^

Second, increases in out-of-state tele-mental health care utilization by new patients, if any, were smaller in magnitude to those by established patients. This suggests that the relationship waiver is not particularly effective in enhancing access to health care for new patients, as intended by policymakers. Inertia in out-of-state telehealth provision following licensure waiver expiration may also be present when waivers are introduced, potentially causing delayed out-of-state use by new patients.^9^ Barriers to accessing mental health care for new patients persist due to a nationwide shortage of providers, with no state presenting a surplus to compensate.^22^ Policy redesign should address regulatory barriers to establishing new patient–physician relationships remotely. These results also support the view that out-of-state telehealth is better suited for continuity of care as opposed to new patient enrollment, even for a population of nonelderly, commercially insured patients.^11^ Reliance on telehealth alone via the relationship waiver is likely insufficient to address the large treatment gaps for new patients.

Third, we found significant heterogeneity across MSAs by level of urbanization.^23^ Following relationship waivers, we document increased out-of-state tele-mental health care by new patients in rural 3-digit zip codes of MSAs neighboring an out-of-state major urban center (Chicago MSA). However, no increase in out-of-state tele-mental health care was observed for new patients in urban 3-digit zip codes of MSAs with an in-state major urban center (St. Louis MSA), suggesting a lower effectiveness of relationship waivers in MSAs with sufficient in-state mental health providers. Decreased utilization by Missouri patients may be attributed to the relative scarcity of out-of-state specialists relative to Illinois patients who have had access to out-of-state specialists through the IMLC. Pre-existing regulation accommodating cross-state health care such as the IMLC may better facilitate expansions in out-of-state telehealth utilization than emergency relationship waivers. Legislators should therefore consider further ways to streamline licensure coordination across states to enhance current tele-mental health care accessibility and boost readiness for future PHEs. In the Davenport MSA, where there is no cross-state variation in IMLC participation, the decreased tele-mental health care utilization of Iowa patients may be attributed to heavy reliance of Illinois patients to out-of-state providers for mental health services (Appendix Table A2). These findings indicate that the relationship waiver has the intended impact only in MSAs where treated patients are also from rural 3-digit zip codes (Chicago MSA) but not where patients are urban (Davenport MSA and St. Louis MSA). There continues to be a digital divide between urban and rural populations in tele-mental health care utilization.^24-27^ Policymakers could use these findings to refine methods of promoting tele-mental health care to rural patients, potentially via targeted outreach or educational campaigns that emphasize its benefits. More investments in rural digital infrastructure are necessary to overcome access barriers from limited availability of high-speed internet but may not be sufficient to establish equitable tele-mental health care access.^28^

Fourth, our findings support the notion that IMLC participation may provide sufficient baseline access to out-of-state tele-mental health care, rendering licensure waivers less important. Still, IMLC participation does not diminish the potential of relationship waivers, which may still be effective in accommodating transition of out-of-state mental health care to a virtual modality in MSAs where both states participate in the IMLC (Davenport MSA).^23,26,27^

### Study limitations

This study is subject to several study limitations. The empirical analysis may be confounded by PHE-related behavioral changes due to shifting patient preferences in favor of telehealth, social-distancing policies, or increased demand for mental health services that may introduce an upward bias in our estimates, thereby overstating the importance of waivers for out-of-state tele-mental health care.^29,30^ Second, we cannot verify the physical location of patients during telehealth visits.^31^ To address some of these concerns, we minimized the influence of local health shocks using a border discontinuity design that compares localities within the same MSA, thereby holding some of those confounders constant. Third, our data may not be representative of the US population given its focus on Midwestern states and the COVID-19–diagnosed patient sample. To the extent that patient preferences, licensure regulations, mental health care needs, and access to high-speed internet vary by geography and incidence of COVID-19, other US localities may be impacted differently by licensure and relationship waivers. Nonetheless, our sample encompasses a diverse population of patients from urban and rural areas that vary in population size, COVID-19 infection rates, and IMLC participation, which instills confidence in the generalizability of our results. Fourth, we did not assess the impact of these policy changes on spending, which may be substantial given changes in telehealth reimbursement practices.^32^ Changes in telehealth and overall utilization may very well track closely with costs.^13,14^

## Conclusion

This evaluation shows that licensure and relationship waivers do not result in increased out-of-state tele-mental health care as a share of all mental health visits overall, but instead enable transition of previously in-person out-of-state health care of established patients to a virtual modality. One goal of telehealth is to expand access to mental health treatment access to a new population of patients. These results suggest that these policy changes may have limited effectiveness in attracting new patients, yet efficiently maintain care continuity for established patients. State medical boards could leverage these insights to reassess regulatory barriers hindering tele-mental health care access by new patients, including simplifying the process in establishing patient–physician relationships via telehealth, and enhancing salience of relationship waivers through outreach. Potential avenues for future research include exploring the long-term effects of telehealth policy changes and the role of health care provider attitudes toward tele-mental health care or MSAs in other US Census regions with similar cross-state variation as the Portland or the D.C. MSAs.

## Supplementary Material

qxae026_Supplementary_Data
