# Supplementary material for: Association of licensure and relationship requirement waivers with out-of-state tele-mental health care, 2019–2021
Source: Health Aff Sch. 2024 Feb 28;2(4):qxae026. doi: 10.1093/haschl/qxae026 (PMC11034529; doi:10.1093/haschl/qxae026)
Supplement: qxae026_Supplementary_Data [file qxae026_supplementary_data.zip › Supplementary Materials CLEAN.docx]

**Supplementary Appendix**

**Appendix Table A1: Additional Data Information**

We use the Change Healthcare dataset from the COVID-19 Research Database, a research and public health consortium led by HHS Technology Group and its database partners dedicated to expanding our understanding surrounding COVID-19 by providing real-time claims data and software to enable COVID-19 related research. Change Healthcare is the largest health insurance claims clearinghouse with a nationwide network of 900,000 providers, 5,500 hospitals nationally, 2,100 payers, 33,000 pharmacies, and 600 labs processing nearly 50% of all commercial claims in the United States. The Change Healthcare dataset was selected for three reasons: 1) its comprehensive reach and nationwide coverage ensures that out-of-state telehealth utilization is sufficiently captured while retaining information on age, gender and insurance status, 2) the granular geographic identifiers available at the 3-digit zip code level allow us to pursue a border discontinuity research design by comparing cross-state patients within the same metropolitan statistical area (MSA), 3) its daily time frequency and immediate availability of medical encounters a single day after claims processing, which makes this dataset well-suited to study the rapidly-changing telehealth policy environment at a granular level.

The Change Healthcare data has separate files for claims and services. Each observation in the claims file denotes a unique visit, and includes information on the visit location, date, insurance status, and limited patient demographic information. The service file contains, for each unique visit, a list of CPT codes which delineate the set of procedures administered at the visit. After applying our sample inclusion criteria to the raw data, we merge these two files using the unique claim number. Because services are nested in a claim, we collapse the services to the claim level and in doing so characterize the visit by location (in-state or out-of-state) and modality (telehealth or in-person). We collapse the data a second time to the patient-month level so as to build a longitudinal dataset that tracks the number of mental health visits by modality and location for each patient in a given month, which serves as our unit of analysis.

We use three measures of out-of-state telehealth utilization. First, out-of-state telehealth utilization as a share of total mental health visits is the most comprehensive measure of mental health utilization in this study by virtue of incorporating in-person visits and in-state, telehealth visits in its denominator, too. Second, out-of-state telehealth utilization as a share of telehealth mental health visits serves as a proxy of cross-state mental health provider switching for telemental care. As a consequence of excluding in-person visits, it may be prone to differential changes in in-state and out-of-state telemental care. Third, out-of-state telehealth utilization as a share of out-of-state mental health visits, similarly, captures mental health provider modality switching but exclusively for out-of-state care. Thus, it is the outcome measure most likely to capture the impact of policy changes affecting out-of-state care.

We restrict our attention to diagnoses under ICD-9/-10 codes F20-F29, F30-F39, F40-F48, F50-F59, F60-F69, F80-F89, F90-F98 and F99 and outpatient visits recorded using CPT codes 9402, 9403, 9408, 9409, 9411-9413, 9419, 9431, 9434, 9436-9439, 9441-9444, 9449, 9451, 9452, 9455, 9459, 90785, 90791, 90792 90801, 90802, 90804, 90806, 90807, 90808, 90810-90815, 90820, 90825, 90830, 90832-90840, 90845-90849, 90853, 90855, 90857, 90862, 90872, 90880, 90885, 90887, 90889, 90899, 90902, 96100-96103, 96110, 96111, 97003. 97004, 98966-98968, 99078, 99201-99205, 99211-99215, 99241-99245, 99371, 99404, 99411, 99412, 99420, 99487, 99489, 99490, 99510, 99843, 0360T, 0361T, 0368T, 0371T-0374T, 98960-98962, 99341-99344, 99349, 99350, 99382-99387, 9939299397, 99401-99404, 99441-99443, and HCPCS codes G0076-G0086, G0092, G0176, G0177, G0438, G0439, G0442, G0446, G0447, G0444, G0468, G0506, G0513, G0514, G2025, G2077-G2080, H0001, HO; H0001, TS; H0002, H0004, H0004, HQ; H0004, HR; H0023-H0025, H0030, H0031, H0031, HA; H0031, HM; H0031, HN; H0031, HO; H0031, TS; H0032, H0032, HK; H0032, TS; H0034, H0038, H0046, H0046, HE; H1011, H2000, H2000, HO; H2000, HP; H2010, H2010, HE; H2010, HM; H2010, HN; H2010, HO; H2010, HP; H2010, HQ; H2012; H2015, H2015, HE; H2015, HN; H2015, HQ; H2016, H2017-H2019, H2019, HM; H2019, HN; H2019, HO; H2019, HQ; H2019, HR; H2020, H2023, H2024, H2027, H2030-H2032, H2037, Q3014, S0280, S0281, S3005, S5100-S5102, S9110, S9127, S9454, S9480, S9482, T1007, SA; T1007, TS; T1007, U8; T1015, T1015, HE; T1017, T1017, HA; T1017, HB; T1017, HK; T1017, TL; T1023, T1023, HE; T1024, T1027, T1040, T1041, T2012-T2015, T2018-T2023; Z0001-Z0003.

| **Appendix Table A2: Share of Total & Telehealth Out-of-State Mental Health Visits by 3-digit Zip Code, 2019-2021** | | | | | | | | | |
| --- | --- | --- | --- | --- | --- | --- | --- | --- | --- |
| State | MSA | County | Population | Living in Urban Blocks (in %) | COVID-19 Infections (per 100k) | Zip Code | Any Modality OOS (in %) | Telehealth OOS MH (in %) |  |
| IN | Chicago | Jasper | 32,892 | 16.74% | 33,620 | 463 | 10.3277% | 1.0257% |  |
|  | Chicago | Lake | 498,932 | 95.46% | 29,454 | 464 | 20.9749% | 1.3483% |  |
|  | Chicago | Jasper | 32,892 | 16.74% | 33,620 | 479 | 58.5985% | 6.4118% |  |
| IA | Davenport | Scott | 174,596 | 86.72% | 28,062 | 527 | 6.8420% | 0.5955% |  |
|  | Davenport | Scott | 174,596 | 86.72% | 28,062 | 528 | 6.8611% | 0.7604% |  |
| IL | Chicago | Cook | 5,261,249 | 99.95% | 29,076 | 600 | 1.3396% | 0.1029% |  |
|  | Chicago | Cook | 5,261,249 | 99.95% | 29,076 | 601 | 2.5503% | 0.1166% |  |
|  | Chicago | Cook | 5,261,249 | 99.95% | 29,076 | 602 | 1.3140% | 0.4987% |  |
|  | Chicago | Cook | 5,261,249 | 99.95% | 29,076 | 603 | 2.5552% | 0.3952% |  |
|  | Chicago | Cook | 5,261,249 | 99.95% | 29,076 | 604 | 3.2805% | 0.2288% |  |
|  | Chicago | Cook | 5,261,249 | 99.95% | 29,076 | 605 | 1.5470% | 0.1290% |  |
|  | Chicago | Cook | 5,261,249 | 99.95% | 29,076 | 606 | 1.2306% | 0.1385% |  |
|  | Chicago | Cook | 5,261,249 | 99.95% | 29,076 | 607 | 0.5335% | 0.0933% |  |
|  | Chicago | Cook | 5,261,249 | 99.95% | 29,076 | 608 | 0.7859% | 0.0535% |  |
|  | Chicago | Kankankee | 107,250 | 71.60% | 34,217 | 609 | 2.9050% | 0.2230% |  |
|  | Davenport | Henry | 49,172 | 50.20% | 32,327 | 612 | 65.2410% | 2.3323% |  |
|  | Davenport | Henry | 49,172 | 50.20% | 32,327 | 614 | 15.5807% | 0.6444% |  |
|  | St. Louis | Jersey | 21,495 | 40.59% | 36,598 | 620 | 13.8740% | 0.9510% |  |
|  | St. Louis | Clinton | 36,913 | 19.78% | 39,004 | 622 | 28.1362% | 1.2227% |  |
|  | St. Louis | Macoupin | 44,845 | 34.48% | 35,757 | 626 | 1.2867% | 0.0926% |  |
| MO | St. Louis | Franklin | 104,853 | 45.01% | 30,027 | 630 | 2.3174% | 0.4220% |  |
|  | St. Louis | St. Louis | 1,003,056 | 100.00% | 29,358 | 631 | 3.5366% | 0.5849% |  |
|  | St. Louis | St. Charles | 406,385 | 94.34% | 29,525 | 633 | 2.8082% | 0.2690% |  |
| WI | Chicago | Kenosha | 169,184 | 86.92% | 33,491 | 531 | 15.6309% | 0.8689% |  |

Abbreviations: MH: mental health; OOS: out-of-state

Notes: This table presents the percentage of total mental health visits that are out-of-state, regardless of modality,and out-of-state via telehealth by zip code over the sample period 2019-2021. Numbers are computed by aggregating visits by modality and location to the zip code level and then computing percentages. The sample includes 2,029,470 distinct patients. Additionally, population, share of individuals living in urban block and COVID-19 infection rates at the county level are shown. COVID-19 infection rates represent the historical number of cases in the county population per 100,000 individuals from March 2020 until March 2023.

*Source*: Authors analysis of the Change Healthcare dataset provisioned by the COVID-19 Research Database, 2019-2021. County-level 2020 Census Urban and Rural Information for the U.S., Puerto Rico, and Island Areas sorted by state and county FIPS codes, Johns Hopkins University COVID-19 Tracker.

**Appendix Figure A1: Inclusion-Exclusion Criteria Flow Diagram**


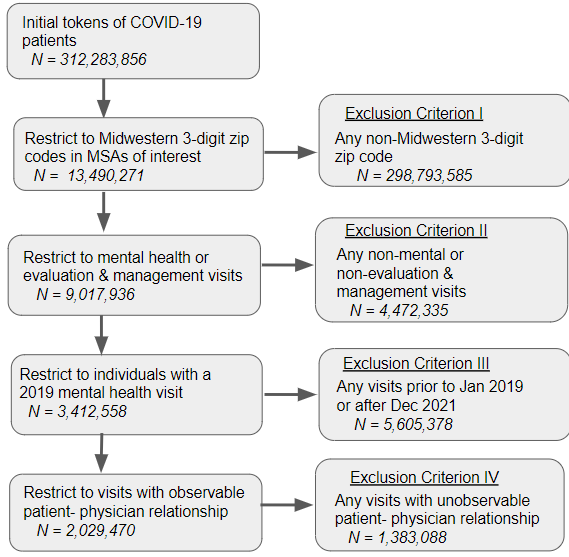


*Source*: Authors analysis of the Change Healthcare dataset provisioned by the COVID-19 Research Database, 2019-2021

**Appendix Figure A2: Policy Variation Timeline**

Prior to the PHE, mental health practitioners are required to hold a license from the medical board of the patient's state in order to provide out-of-state services. An exception to this requirement is permitting the practitioner to serve patients who reside in a state that participates in a licensure compact, such as the Interstate Medical Licensure Compact, provided the practitioner is also based in a participating state. Generally, establishing a patient-physician relationship via telehealth is permitted only in cases where the standard of care may not require an in-person encounter. Further limitations to establishing such a relationship include restrictions to the setting a patient may be during a tele-encounter or the modalities that can be used.^[[1]](#footnote-1)^ All study states introduce in-state licensure requirement waivers in March 2020. Iowa is the first to introduce in-state licensure requirement waivers on March 16 followed by Missouri on March 18, Illinois and Indiana on March 19 and Wisconsin on March 27, 2020. These licensure waivers are in place till the end of our study period on December 31, 2021 for all states but Wisconsin where they expire on June 10, 2020 and are re-introduced from October 1, 2020 till April, 4, 2021. Other emergency policy changes include relaxation of HIPAA enforcement, audiovisual modality and originating site requirements to access telehealth. As these coincide with licensure waivers, we group them under the umbrella term licensure waivers to reflect the relative importance of the in-state licensure requirement waivers stemming from their broad applicability to narrower changes impacting either modality or place of service. This bundle of policy variation excludes changes in payment parity. Our treatment group consists of patients in Indiana, Iowa, Missouri, and Wisconsin where pre-existing patient-physician relationship requirement waivers are additionally adopted until the end of our study period with the exception of Wisconsin. These relationship waivers are introduced concurrently with the licensure waivers in all states but Missouri where they become effective only after July 16, 2020. Our control group consists of patients residing in Illinois. As aforementioned, Illinois enacts only the in-state licensure requirement waiver on March 19, 2020 while keeping the requirement for a pre-existing patient-physician relationship intact. This policy variation in waiver enactment (panel A) and baseline IMLC participation (panel B) are summarized in the plots below:

**Panel A: Telehealth Waiver Variation**


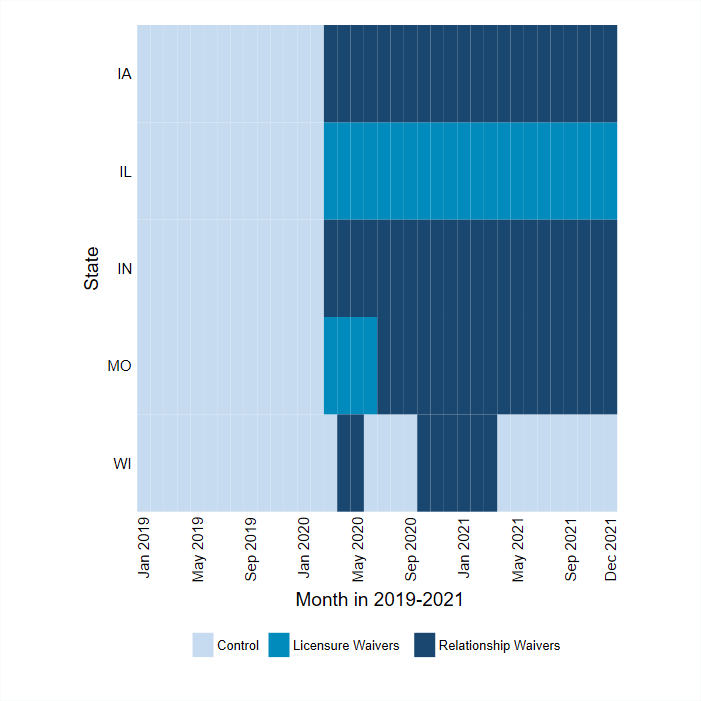


*Source*: Federation of State Medical Boards. “US states and territories modifying requirements for telehealth in response to COVID-19”, accessed November 1, 2023 <https://www.fsmb.org/siteassets/advocacy/pdf/states-waiving-licensure-requirements-for-telehealth-in-response-to-covid-19.pdf>

**Panel B: Interstate Medical Licensure Compact Adoption**


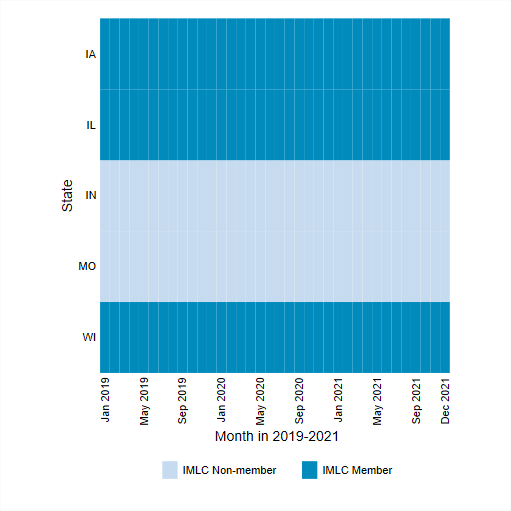


*Source*: Interstate Medical Licensure Compact, “Participating States”, accessed November 1, 2023, <https://www.imlcc.org/participating-states/>

**Appendix Table A3: Regression Specification & Policy Variation Timeline**

Table 2 Specification:

$$Y_{izst}=\beta_{0}+\beta_{1}{Post}_{t}+\beta_{2}{Treat}_{s}+ \beta_{3}{Post}_{t}*{Treat}_{s}+\alpha_{z}+\tau_{t}+\boldsymbol{X'}\boldsymbol{\beta}+\varepsilon_{izst}$$

Where $i$ indexes the individual, $z$ the zip code, $s$ the state and $t$ the month.

Outcome $Y_{izst}$ is out-of-state telehealth mental healthcare (MH) visits as a share of MH visits in column (1), new patient out-of-state telehealth MH visits as a share of new patient MH visits in column (2), established patient out-of-state telehealth MH visits as a share of established patient MH visits in column (3), out-of-state telehealth MH visits as a share of telehealth MH visits in column (4), new patient out-of-state telehealth MH visits as a share of new patient telehealth MH visits in column (5), established patient out-of-state telehealth MH visits as a share of established patient telehealth MH visits in column (6), out-of-state telehealth MH visits as a share of out-of-state MH visits in column (7), new patient out-of-state telehealth MH visits as a share of new patient out-of-state MH visits in column (8), established patient out-of-state telehealth MH visits as a share of established patient out-of-state MH visits in column (9).

${Post}_{t}$ is an indicator variable taking values equal to 1 from April 2020 onwards; 0, otherwise. ${Treat}_{s}$ is an indicator for states Indiana, Iowa, Missouri and Wisconsin. The key parameter of interest in the difference-in-differences model is the interaction of the post-licensure waivers indicator with the pre-existing relationship requirement indicator ${Post}_{t}*{Treat}_{s}$ . Zip code fixed effects are denoted by $\alpha_{z}$ and year-month fixed effects are denoted by $\tau_{t}$. Individual-level covariates are captured in vector $\boldsymbol{X}$ and include gender, age, and insurance status. Standard errors are clustered at the state level. Regression information can be found in Table 2 in the Manuscript.  In an alternative regression model using state fixed effects and 3-digit zip code level covariates we adjust by total population, adult population, percent white, percent black, percent Asian, percent other, percent Hispanic, percent female, percent male, poverty rate, unemployment rate, per capital income, high school graduation rate, percent older than 65, percent under 18, percent disabled, and percent single parent household (Appendix Table A5).

Table 3 Specification:

$$Y_{izst}=\beta_{0}+\beta_{1}{Post}_{t}+\beta_{2}{Treat}_{s}+ \beta_{3}{Post}_{t}*{Treat}_{s}+\alpha_{z}+\tau_{t}+\boldsymbol{X'}\boldsymbol{\beta}+\varepsilon_{izst}$$

Where $i$ indexes the individual, $z$ the zip code, $s$ the state and $t$ the month. In columns (1)-(3), individual $i$ is a resident of the Chicago-Naperville-Joliet MSA, in columns (4)-(6) the individual is a resident of the Davenport-Moline-Rock Island MSA and in columns (7)-(9) the individual is a resident of the St. Louis MSA.

Outcome $Y_{izst}$ is out-of-state telehealth mental healthcare (MH) visits as a share of MH visits, out-of-state telehealth MH visits as a share of telehealth MH visits, and out-of-state telehealth MH visits as a share of out-of-state MH visits in columns (1), (4), and (7), respectively. Under columns (2), (5), and (8), outcome $Y_{izst}$ is new patient out-of-state telehealth MH visits as a share of new patient MH visits, new patient out-of-state telehealth MH visits as a share of new patient telehealth MH visits, and new patient out-of-state telehealth MH visits as a share of new patient out-of-state MH visits, respectively. Under columns (3), (6), and (9), outcome $Y_{izst}$ is established patient out-of-state telehealth MH visits as a share of established patient MH visits, established patient out-of-state telehealth MH visits as a share of established patient telehealth MH visits, and established patient out-of-state telehealth MH visits as a share of established patient out-of-state MH visits, respectively.

${Post}_{t}$ is an indicator variable taking values equal to 1 from April 2020 onwards; 0, otherwise. ${Treat}_{s}$ is an indicator for states Indiana, Iowa, Missouri and Wisconsin. The key parameter of interest in the difference-in-differences model is the interaction of the post-licensure waivers indicator with the relationship waiver indicator ${Post}_{t}*{Treat}_{s}$. Zip code fixed effects are denoted by $\alpha_{z}$ and year-month fixed effects are denoted by $\tau_{t}$. Individual-level covariates are captured in vector $\boldsymbol{X}$ and include gender, age, and insurance status. Standard errors are clustered at the state level. Regression information can be found in Table 3 of the Manuscript.

| **Appendix Table A4: Common CPT Codes (number of visits), 2019-2021** | | | | | | | | |
| --- | --- | --- | --- | --- | --- | --- | --- | --- |
| Code | Code Description | Count | Percent |  | Code | Code Description | Count | Percent |
|  |  |  |  |  |  |  |  |  |
| In-state in-person | | | |  | In-state telehealth | | | |
|  |  |  |  |  |  |  |  |  |
| 99213 | Est. patient visit (15 min) | 5,756,586 | 24.57% |  | 90837 | Psychotherapy (60 min) | 1,108,526 | 26.59% |
| 99214 | Est. patient visit (25 min) | 5,243,669 | 22.38% |  | 99213 | Est. patient visit (15 min) | 887,657 | 21.29% |
| T1015 | Clinic visit at FQHC or RHC | 1,729,495 | 7.38% |  | 99214 | Est. patient visit (25 min) | 618,308 | 14.83% |
| 90837 | Psychotherapy (60 min) | 1,562,145 | 6.67% |  | 90834 | Psychotherapy (45 min) | 334,487 | 8.02% |
| 99212 | Est. patient visit (10 min) | 767,478 | 3.28% |  | T1015 | Clinic visit at FQHC or RHC | 283,518 | 6.80% |
| 36415 | Venipuncture, Routine | 758,859 | 3.24% |  | 99442 | Phone E/M by Physician, 11-20 min | 180,072 | 4.32% |
| 99203 | New patient visit (30 min) | 722,653 | 3.08% |  | 99212 | Office/outpatient visit, est | 169,338 | 4.06% |
| 99204 | New patient visit (45 min) | 577,976 | 2.47% |  | 90832 | Psychotherapy (30 min) | 129,428 | 3.10% |
| 90471 | Immunization Administration | 520,730 | 2.22% |  | 99490 | Chronic care management | 114,524 | 2.75% |
| 90834 | Psychotherapy (45 min) | 516,265 | 2.20% |  | 90833 | Psychotherapy (30 min) with E/M | 90,675 | 2.17% |
| Total |  | 23,433,127 |  |  | Total |  | 4,169,617 |  |
| Out-of-state in-person | | | |  | Out-of-state telehealth | | | |
|  |  |  |  |  |  |  |  |  |
| 99213 | Est. patient visit (15 min) | 440,404 | 24.57% |  | 99213 | Est. patient visit (15 min) | 60,625 | 21.94% |
| 99214 | Est. patient visit (25 min) | 431,273 | 22.38% |  | 99214 | Est. patient visit (25 min) | 42,095 | 15.23% |
| 99203 | New patient visit (30 min) | 85,795 | 7.38% |  | 90837 | Psychotherapy (60 min) | 37,826 | 13.69% |
| G0483 | Drug test | 68,150 | 6.67% |  | 99441 | Telephone E/M (5-10 min) | 24,591 | 8.90% |
| 99204 | New patient visit (45 min) | 61,074 | 3.28% |  | 99442 | Telephone E/M (20-30 min) | 22,921 | 8.29% |
| 36415 | Venipuncture, Routine | 55,225 | 2.99% |  | 90834 | Psychotherapy (45 min) | 17,117 | 6.19% |
| 99212 | Est. patient visit (10 min) | 54,967 | 2.98% |  | 99212 | Est. patient visit (10 min) | 12,185 | 4.41% |
| 99343 | New patient home visit | 50,407 | 2.73% |  | 99443 | Phone E/M service by Physician, 21-30 min | 11,750 | 4.25% |
| 90837 | Psychotherapy (60 min) | 49,651 | 2.69% |  | 99201 | New patient visit (10 min) | 5,545 | 2.01% |
| 80307 | Drug screen, single analyte | 45,695 | 2.47% |  | 99490 | Chronic care management | 4,701 | 1.70% |
| Total |  | 23,433,127 |  |  | Total |  | 4,169,617 |  |

*Notes*: This table presents the most common CPT codes for four categories of modality and physician location. For each category, we present the ten most common codes by number of visits. We also include the total number of visits for each category. Please note that "Est" stands for established, "E/M" for Evaluation and Management.

*Source*: Authors analysis of the Change Healthcare dataset provisioned by the COVID-19 Research Database, 2019-2021

**Appendix Figure A3: Coefficient Plot of Change in Out-of-state Tele-mental Healthcare Visits After Licensure and Relationship Waivers**


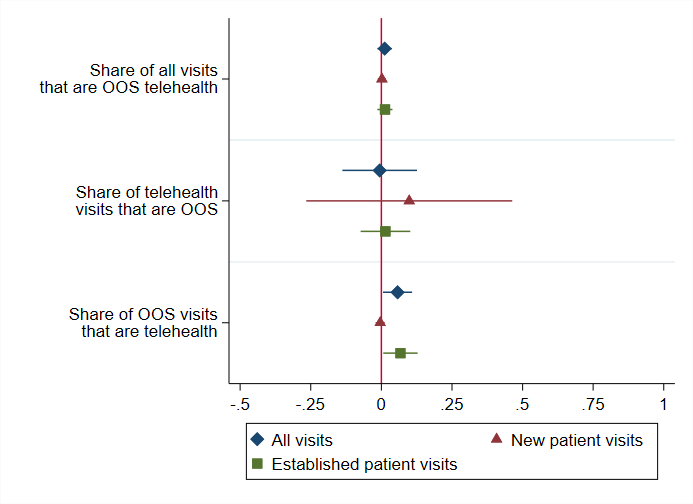


*Notes*: This figure depicts the difference-in-differences effect of pre-existing relationship waivers on mental health visits. The top row of the y-axis panel lists the share of telehealth visits that are out-of-state (OOS), the middle row the share of telehealth visits that are out-of-state and the bottom row the share of out-of-state visits that are telehealth. The first grouping, “Share of all visits that are OOS telehealth”, depicts coefficient estimates for all visits (blue diamonds), new patient visits (red triangles), and established patient visits (green squares) for outcome OOS telehealth / any. The second grouping, “Share of telehealth visits that are OOS”, depicts coefficient estimates for all visits, new patient visits, and established patient visits for outcome OOS telehealth / telehealth. The third and final grouping, “Share of OOS visits that are telehealth”, depicts coefficient estimates for all visits, new patient visits, and established patient visits for out-of-state tele-mental healthcare visits as a share of out-of-state mental healthcare visits. Bars represent 95% confidence intervals.

*Source*: Authors analysis of the Change Healthcare dataset provisioned by the COVID-19 Research Database, 2019-2021

| **Appendix Table A5: Change in Out-of-state Tele-mental Healthcare Visits After Licensure and Relationship Waivers** | | | | | | | | | | | |
| --- | --- | --- | --- | --- | --- | --- | --- | --- | --- | --- | --- |
|  | Share of total visits | | |  | Share of telehealth visits | | |  | Share of out-of-state visits | | |
|  | (1) | (2) | (3) |  | (4) | (5) | (6) |  | (7) | (8) | (9) |
|  | All patient, visits (95% CI) | New patient, visits (95% CI) | Established patient, visits (95% CI) |  | All patient, visits (95% CI) | New patient, visits (95% CI) | Established patient, visits (95% CI) |  | All patient, visits (95% CI) | New patient, visits (95% CI) | Established patient, visits (95% CI) |
| Post | 0.0084** | 0.0457** | 0.0043* |  | -0.1232** | -0.3687*** | -0.0515* |  | 0.1411* | 0.3228* | 0.0901** |
|  | (0.0029, 0.0139) | (0.0147, 0.0767) | (0.0012, 0.0074) |  | (-0.1779, -0.0685) | (-0.4191, -0.3183) | (-0.0919, -0.0111) |  | (0.0147, 0.2675) | (0.0776, 0.5680) | (0.0268, 0.1534) |
| Treat | -0.0335*** | -0.0069*** | -0.0361*** |  | -0.4699*** | -0.2156** | -0.4759*** |  | -0.0373** | 0.0983*** | -0.0505** |
|  | (-0.0384, -0.0286) | (-0.0096, -0.0042) | (-0.0411, -0.0311) |  | (-0.5249, -0.4149) | (-0.3644, -0.0668) | (-0.5142, -0.4376) |  | (-0.0558, -0.0188) | (0.0636, 0.1330) | (-0.0777, -0.0233) |
| Post x Treat | 0.0118 | 0.002 | 0.0129 |  | -0.0045 | 0.0999 | 0.0157 |  | 0.0575** | -0.0041 | 0.0678** |
|  | (-0.0064, 0.0300) | (-0.0084, 0.0124) | (-0.0061, 0.0319) |  | (-0.0974, 0.0884) | (-0.1573, 0.3571) | (-0.0458, 0.0772) |  | (0.0212, 0.0938) | (-0.0155, 0.0073) | (0.0249, 0.1107) |
|  |  |  |  |  |  |  |  |  |  |  |  |
| State FE | Yes | Yes | Yes |  | Yes | Yes | Yes |  | Yes | Yes | Yes |
| Zip FE | No | No | No |  | No | No | No |  | No | No | No |
| Baseline month | March | March | March |  | March | March | March |  | March | March | March |
| Dep. var mean | 0.0005 | 0.0017 | 0.0004 |  | 0.108 | 0.6484 | 0.0727 |  | 0.0084 | 0.019 | 0.0063 |
| Dep. var mean - treated state | 0.001 | 0.0028 | 0.0007 |  | 0.207 | 0.6977 | 0.1477 |  | 0.0123 | 0.027 | 0.0094 |
| N | 9,144,952 | 1,231,017 | 8,239,083 |  | 723,290 | 32,272 | 696,474 |  | 661,191 | 114,988 | 567,391 |

Abbreviation: CI: confidence intervals; N: observations

*Notes*: This table presents the difference-in-differences regression estimates (95% CI in parentheses) of the association of licensure and relationship waivers with out-of-state tele-mental healthcare. The sample includes patients in Illinois, Indiana, Iowa, Missouri, and Wisconsin over the years 2019-2021. For columns (1)-(3), the outcome is the share of all mental visits that are out-of-state telehealth, for (4)-(6), the share of telehealth visits that are out-of-state , and for (7)-(9), the share of out-of-state visits that are telehealth. Columns (1), (4), and (7) consider all patient visits, (2), (5), and (8) consider new patient visits, and (3), (6), and (9) consider established patient visits. In all relationship-specific columns, denominators of the outcome variables are themselves relationship-specific; e.g., the outcome for column (2) is the total number of new patient out-of-state telehealth mental health visits divided by the total number of new patient mental health visits. Post is an indicator variable taking values equal to 1 from April 2020 onwards; 0, otherwise. Treat is an indicator for Indiana, Iowa, Missouri and Wisconsin. All regressions include state and month fixed effects, zip-code covariates (total population, adult population, percent white, percent black, percent Asian, percent other, percent Hispanic, percent female, percent male, poverty rate, unemployment rate, per capital income, high school graduation rate, percent older than 65, percent under 18, percent disabled, and percent single parent household), and individual covariates (gender, age, and insurance status).

*Source*: Authors analysis of the Change Healthcare dataset provisioned by the COVID-19 Research Database, 2019-2021

| **Appendix Table A6: Change in Out-of-state Tele-mental Healthcare Visits After Licensure and Relationship Waivers** | | | | | | | | | | | |
| --- | --- | --- | --- | --- | --- | --- | --- | --- | --- | --- | --- |
|  | Share of total visits | | |  | Share of telehealth visits | | |  | Share of out-of-state visits | | |
|  | (1) | (2) | (3) |  | (4) | (5) | (6) |  | (7) | (8) | (9) |
|  | All patient, visits (95% CI) | New patient, visits (95% CI) | Established patient, visits (95% CI) |  | All patient, visits (95% CI) | New patient, visits (95% CI) | Established patient, visits (95% CI) |  | All patient, visits (95% CI) | New patient, visits (95% CI) | Established patient, visits (95% CI) |
| Post | 0.0085** | 0.0456** | 0.0044** |  | -0.1154*** | -0.3942*** | -0.0487** |  | 0.1412* | 0.3217* | 0.0906** |
|  | (0.0032, 0.0138) | (0.0145, 0.0767) | (0.0015, 0.0073) |  | (-0.1336, -0.0972) | (-0.4046, -0.3838) | (-0.0791, -0.0183) |  | (0.0150, 0.2674) | (0.0749, 0.5685) | (0.0277, 0.1535) |
| Treat | 0.001 | 0.003 | 0.0008 |  | 0.1644 | 0.1416 | 0.1273 |  | -0.0098 | 0.0016 | -0.0116 |
|  | (-0.0074, 0.0094) | (-0.0013, 0.0073) | (-0.0082, 0.0098) |  | (-0.0469, 0.3757) | (-0.1544, 0.4376) | (-0.0213, 0.2759) |  | (-0.0255, 0.0059) | (-0.0045, 0.0077) | (-0.0314, 0.0082) |
| Post x Treat | 0.0117 | 0.0023 | 0.0126 |  | -0.0249 | 0.1511 | 0.0071 |  | 0.0578** | -0.0011 | 0.0675** |
|  | (-0.0063, 0.0297) | (-0.0079, 0.0125) | (-0.0062, 0.0314) |  | (-0.2407, 0.1909) | (-0.1749, 0.4771) | (-0.1442, 0.1584) |  | (0.0229, 0.0927) | (-0.0129, 0.0107) | (0.0259, 0.1091) |
| State FE | No | No | No |  | No | No | No |  | No | No | No |
| Zip FE | Yes | Yes | Yes |  | Yes | Yes | Yes |  | Yes | Yes | Yes |
| Baseline month | February | February | February |  | February | February | February |  | February | February | February |
| Dep. var mean | 0.0004 | 0.0015 | 0.0002 |  | 0.1946 | 0.7696 | 0.1157 |  | 0.0062 | 0.0177 | 0.0038 |
| Dep. var mean - treated state | 0.0007 | 0.0026 | 0.0005 |  | 0.2683 | 0.7581 | 0.1709 |  | 0.0091 | 0.0249 | 0.0058 |
| N | 9,144,952 | 1,231,017 | 8,239,083 |  | 723,290 | 32,272 | 696,474 |  | 661,191 | 114,988 | 567,391 |

Abbreviation: CI: confidence intervals; N: observations

Notes: This table presents the difference-in-differences regression estimates (95% CI in parentheses) of the association of licensure and pre-existing relationship waivers with out-of-state tele-mental healthcare. The sample includes patients in Illinois, Indiana, Iowa, Missouri, and Wisconsin over the years 2019-2021. For columns (1)-(3), the outcome is the share of all mental visits that are out-of-state telehealth, for (4)-(6), the share of telehealth visits that are out-of-state , and for (7)-(9), the share of out-of-state visits that are telehealth. Columns (1), (4), and (7) consider all patient visits, (2), (5), and (8) consider new patient visits, and (3), (6), and (9) consider established patient visits. In all relationship-specific columns, denominators of the outcome variables are themselves relationship-specific; e.g., the outcome for column (2) is the total number of new patient out-of-state telehealth mental health visits divided by the total number of new patient mental health visits. indicator variable taking values equal to 1 from March 2020 onwards; 0, otherwise. Treat is an indicator for Indiana, Iowa, Missouri and Wisconsin. All regressions include zip code and month fixed effects and individual covariates (gender, age, and insurance status) which are omitted from the table. Standard errors are clustered by state.

*Source*: Authors analysis of the Change Healthcare dataset provisioned by the COVID-19 Research Database, 2019-2021

**Appendix Figure A4: Event Study Plot of Change in Out-of-state Tele-mental Healthcare Visits as a Share of All Visits After Relationship Waivers, 2019-2021**


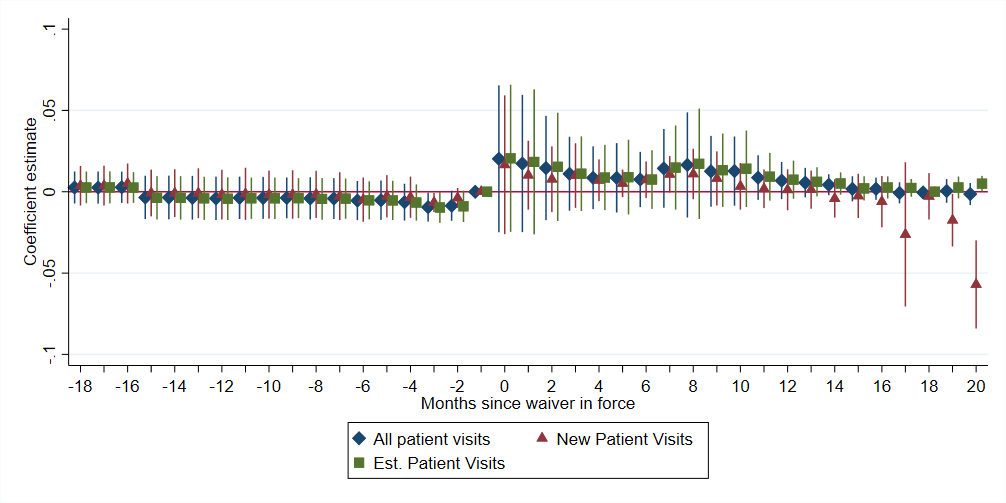


*Notes*: This figure depicts the dynamic difference-in-differences effects of pre-existing relationship waivers on out-of-state tele-mental healthcare visits as a share of all mental health visits. On the horizontal axis is the months since the waiver was implemented, with zero being the month of implementation (April) and the baseline month being one month prior to implementation (March). On the vertical axis is the coefficient estimate for that event-month. Estimates for all patient visits (purple diamonds), new patient visits (red triangles), and established patient visits (green squares) are depicted separately. Bars represent 95% confidence intervals.

*Source*: Authors analysis of the Change Healthcare dataset provisioned by the COVID-19 Research Database, 2019-2021

**Appendix Figure A5: Event Study Plot of Change in Out-of-state Tele-mental Healthcare Visits as a Share of All Tele-mental Healthcare Visits After Relationship Waivers, 2019-2021**


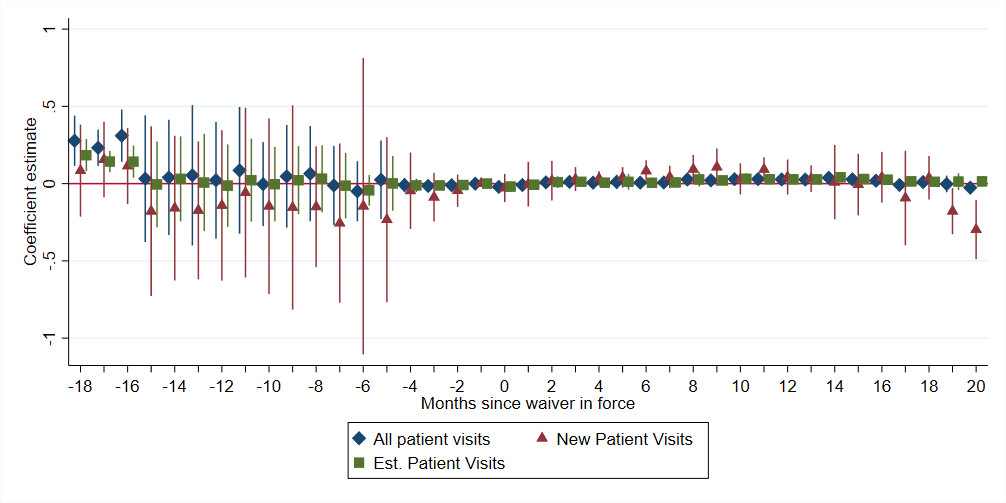


*Notes*: This figure depicts the dynamic difference-in-differences effects of pre-existing relationship waivers on out-of-state tele-mental healthcare visits as a share of all telehealth mental health visits. On the horizontal axis is the months since the waiver was implemented, with zero being the month of implementation (April) and the baseline month being one month prior to implementation (March). On the vertical axis is the coefficient estimate for that event-month. Estimates for all patient visits (purple diamonds), new patient visits (red triangles), and established patient visits (green squares) are depicted separately. Bars represent 95% confidence intervals.

*Source*: Authors analysis of the Change Healthcare dataset provisioned by the COVID-19 Research Database, 2019-2021

**Appendix Figure A6: Event Study Plot of Change in Out-of-state Tele-mental Healthcare Visits as a Share of All Out-of-state Mental Care Visits After Relationship Waivers, 2019-2021**


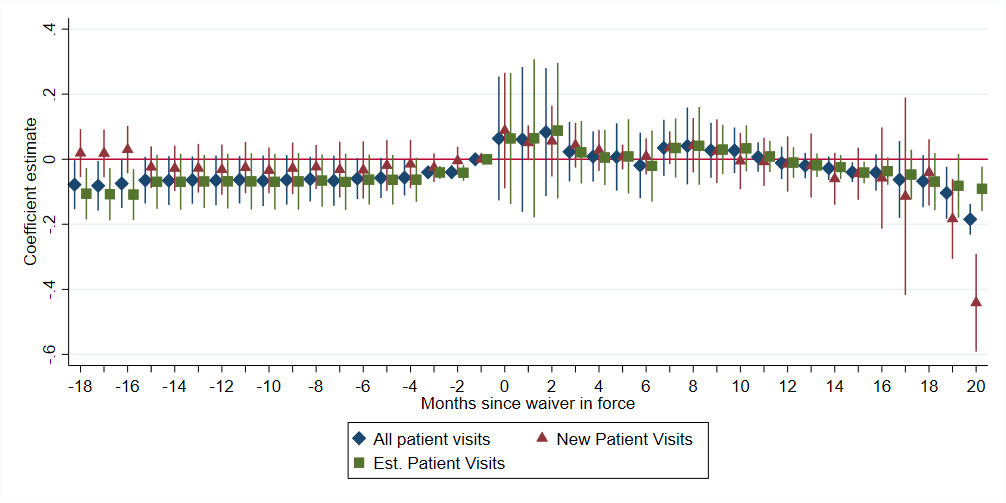


*Notes*: This figure depicts the dynamic difference-in-differences effects of pre-existing relationship waivers on out-of-state tele-mental healthcare visits as a share of all out-of-state mental health visits. On the horizontal axis is the months since the waiver was implemented, with zero being the month of implementation (April) and the baseline month being one month prior to implementation (March). On the vertical axis is the coefficient estimate for that event-month. Estimates for all patient visits (purple diamonds), new patient visits (red triangles), and established patient visits (green squares) are depicted separately. Bars represent 95% confidence intervals.

*Source*: Authors analysis of the Change Healthcare dataset provisioned by the COVID-19 Research Database, 2019-2021

**Appendix Figure A7: Coefficient Plot of Change in Out-of-state Tele-mental Healthcare Visits After Relationship Waivers, 2019-2021 by Age Groups**


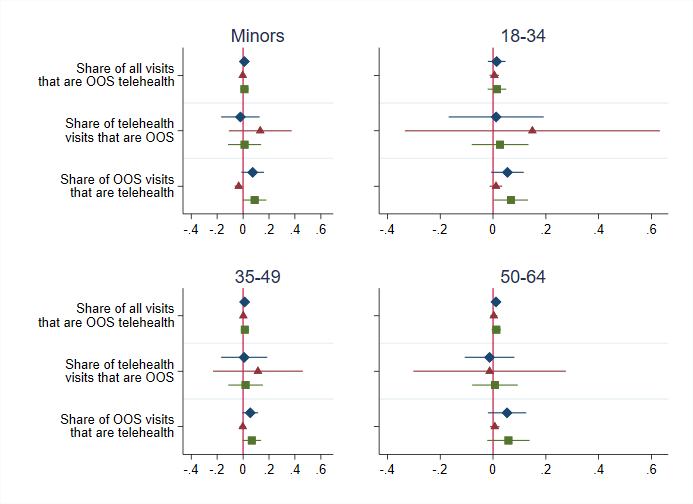


*Notes*: This figure depicts the difference-in-differences effects of pre-existing relationship waivers on the number of out-of-state tele-mental healthcare visits per month by age groups: (i) top left, minors aged 0-17; (ii) top right, aged 18-34; (iii) bottom left, aged 35-49; (iv) bottom right, aged 50-64. The top row of each panel’s y-axis lists the share of telehealth visits that are out-of-state, the middle row the share of telehealth visits that are out-of-state and the bottom row the share of out-of-state visits that are telehealth. The first grouping, “Share of all visits that are OOS telehealth”, depicts coefficient estimates for all visits (purple diamonds), new patient visits (red triangles), and established patient visits (green squares) for outcome OOS telehealth / any. The second grouping, “Share of telehealth visits that are OOS”, depicts coefficient estimates for all visits, new patient visits, and established patient visits for outcome OOS telehealth / telehealth. The third and final grouping, “Share of OOS visits that are telehealth”, depicts coefficient estimates for all visits, new patient visits, and established patient visits for out-of-state tele-mental healthcare visits as a share of all out-of-state mental health visits. Bars represent 95% confidence intervals.

*Source*: Authors analysis of the Change Healthcare dataset provisioned by the COVID-19 Research Database, 2019-2021

1. Advocacy Resource Center, American Medical Association: 50-state survey: Establishment of a patient-physician relationship via telemedicine, accessed 01.28.2024: <https://www.ama-assn.org/system/files/2018-10/ama-chart-telemedicine-patient-physician-relationship.pdf> [↑](#footnote-ref-1)
